# Supplementary material for: Prognostic role of perioperative acid-base disturbances on the risk of Clostridioides difficile infection in patients undergoing cardiac surgery
Source: PLoS One. 2021 Mar 17;16(3):e0248512. doi: 10.1371/journal.pone.0248512 (PMC7968627; doi:10.1371/journal.pone.0248512)
Supplement: Attachment — Submitted filename: Answers to Reviewers.docx [file pone.0248512.s002.docx]

Dear Reviewer of *PLOS ONE*,

Thank you very much for your patience and all the valuable comments and suggestions. I tried my best to address all your concerns. Please find my answers below.

Sincerely yours,

Dariusz Plicner on behalf of the authors.

**1.** Please indicate what type of operations the patients undergo and the clamp and bypass times for these operations. It would be important to know if increased bypass and clamp times have an effect on cdiff operations.

***Ad. 1*** *We performed additional calculations according to your recommendations.*

*The most common surgical procedures performed in the analyzed groups of patients were heart valve surgery and CABG (for CDI patients: 37.8% and 37.1% and for non-CDI patients: 28.0% and 46.5%, respectively). There was no difference between groups in the type of operations (p = 0.448).*

*Patients with CDI had longer cardiopulmonary bypass time than the control group (p = 0.01), but there were no correlations between CDI occurrence and* *aortic cross-clamp time (p = 0.144 ).*

***We added the above findings to the results section (p. 10, lines 176-178, 181-182 and Table 3) and discussion (p. 14, lines 258-260).***

**2.** Having different types of preoperative antibiotics may have an effect on cdiff infections. What is the reasoning for using these different types of antibiotics instead of having the standard. How about post-op antibiotics. At our institution, we usually continue antibiotics for another 3-5 doses post-operatively.
***Ad. 2*** *At our institution, each patient who undergoes cardiac surgery receives periprocedural antimicrobial prophylaxis, based on the first generation of cephalosporins (cefazolin). This is also continued for another 3-5 doses postoperatively. Only in cases of history of allergy to cephalosporins or penicillin, clindamycin was administered.* *There was no difference in the type of perioperative antimicrobial prophylaxis used in the patients with and without CDI (92% vs 94 %, respectively, p = 0.537).*

***In compliance with your suggestions we added this information to the methods section (p. 5, lines 92-94) and results (p. 10, lines 179-180).***

**3.** It is possible to take a look at patients who did not have postoperative infections requiring antibiotics. This way, we can isolate those patients who did not require escalation in antibiotics who developed cdiff. We already know additional or escalating antibiotics is a risk factor for cdiff infections. In the same manner, should we considering doing the same with endocarditis patients.

***Ad. 3*** *Thank you for this valuable comment. It offers a very interesting and broad subject for our next study. We already have a large group of patients collected from 2014. We also believe that this is an important area to study which is why we mentioned this problem in the limitations section (p. 16, lines 292-298).*
